# Effects of dietary vitamins on obesity-related metabolic parameters

**DOI:** 10.1017/jns.2023.30

**Published:** 2023-04-12

**Authors:** Chooi Yeng Lee

**Affiliations:** School of Pharmacy, Monash University Malaysia, Subang Jaya, 47500 Selangor, Malaysia

**Keywords:** Adipogenesis, Anti-inflammatory, Antioxidant, Diet, Obesity, Vitamin

## Abstract

Type 2 diabetes mellitus (T2DM) is one of the leading causes of death worldwide. Genetic factors, some underlying medical conditions, and obesity are risk factors of T2DM. Unlike other risk factors which are non-modifiable, obesity is preventable and usually treatable, and is largely contributed by lifestyle factors. Management of these lifestyle factors may curb the development of T2DM and reduces T2DM prevalence. Dietary vitamins have been recommended as a lifestyle modification intervention to support obesity treatment. Vitamins correlate negatively with body weight, body mass index and body composition. Some of the vitamins may also have anti-adipogenic, anti-inflammatory and antioxidant effects. However, results from pre-clinical and clinical studies of the effects of vitamins on obesity are inconsistent. A clear understanding of the effects of vitamins on obesity will help determine dietary intervention that is truly effective in preventing and treating obesity as well as obesity-related complications including T2DM. This article reviews existing evidences of the effects of vitamin supplementation on obesity and obesity-related metabolic status.

## Introduction

The World Health Organization defines obesity as individuals having body mass index (BMI) equals to or greater than 30 kg/m^2^. Obesity is associated with adipose tissue (AT) dysfunction, which is contributed mainly by adipocyte hypertrophy^(1)^. The adipocyte remodelling induces macrophages infiltration, inflammatory cytokine production and synthesis of collagens that limits adipogenesis, and reduces AT's storage capacity^(2)^, leading to triglyceride accumulation in the liver, heart and around blood vessels^(3)^. Weight loss improves AT dysfunction and its adipogenic effects^(4)^.

Presently, the United States Food and Drug Administration (FDA)-approved pharmacotherapy for weight reduction treatment in non-diabetic patients such as liraglutide promotes weight loss, satiety and insulin secretion, the latter overcomes postprandial hyperglycaemia. Liraglutide, however, does not improve AT dysfunction in subcutaneous tissue or prevent inflammation, and does not improve adipogenesis although it has high homology to native glucagon-like peptide-1 (GLP-1), which was reported to reduce inflammation^(5)^ and increase lipolysis^(6)^.

In a randomised controlled trial (RCT) involving type 2 diabetes (T2DM) patients, 4 months of a once-daily injection of liraglutide effectively reduced body weight, visceral AT and fasting glucose, but the treatment induced inflammation^(7)^. In the study^(7)^, the subcutaneous AT RNA and protein expression of tumour necrosis factor-α (TNF-α) and macrophage chemoattractant protein-1 (MCP-1), and the serum MCP-1 levels were significantly increased after 4 months of treatment. Besides that, the subcutaneous AT expression of peroxisome proliferator-activated receptor-γ (PPARγ) and lipoprotein lipase (LPL) were unchanged after liraglutide treatment. PPARγ is a nuclear transcription factor that up-regulates the expression of genes involved in lipid storage and adipogenesis, whereas LPL, which hydrolyses triglycerides, is a marker of adipocyte differentiation, and it increases with triglyceride accumulation. GLP-1 analogues have generally been reported to cause rebound weight gain once therapies are discontinued^(8)^, and liraglutide is of no exception. Liraglutide treatment beyond 20 weeks may be associated with treatment resistance, and patients regain weight beyond 36 weeks^(9,10)^. Another limitation of liraglutide is that it causes gastrointestinal-related side effects early in the treatment course^(11)^.

Consider the limitation of pharmacotherapies and cost–benefit factors, combining short-term liraglutide with behavioural therapies that promote lifestyle changes was advocated^(5)^. Wharton *et al.*^(12)^ suggested adjunctive pharmacotherapy for weight loss and weight loss maintenance for obese individuals or individuals with BMI equals to or more than 27 kg/m^2^ with adiposity-related complications, to support medical nutrition therapy, physical activity and psychological interventions.

Dietary strategies have been reported for a decade or more as an effective intervention in reducing body weight, where previously, the focus was on macronutrient consumption. Now, contributed by a better understanding of the pathophysiology of obesity as well as the limitation of drug therapies, specific dietary intervention, especially diet with antioxidant and anti-inflammatory properties, is recommended for protection against obesity manifestation^(13)^. The literature on dietary intervention that is available to date is instructive to achieving positive and sustainable treatment outcomes. Accordingly, the management of obesity is ideally consisting of dietary, pharmacological, physical and psychological therapies^(14)^.

This review aimed to give an overview and critical review of presently published studies on the effects of vitamins on obesity. It focuses on presenting the effects of vitamins on obese-related metabolic parameters. This enables a clear understanding of the effects of vitamins on the obese population specifically. Where studies from obese subjects are absent, studies involving patients with T2DM or metabolic syndrome, and results from pre-clinical studies are included and these are stated accordingly in the review. Studies which involved obese subjects still formed the majority of the review. Obesity is the main cause of metabolic syndrome, and both conditions increase the risk for hyperglycaemia, dyslipidemia, T2DM, cardiovascular disease and certain cancer^(13)^. The conditions are closely related where individuals who are obese, or have T2DM or metabolic syndrome could all have dysregulated adipocytokines production, increased inflammatory markers and altered blood glucose, insulin sensitivity and lipid profiles.

## Dietary vitamins

Specific vitamins are deficient in obese individuals, irrespective of age groups. A negative correlation between multivitamins and the occurrence of obesity in children and adolescents was reported^(15)^. In contrast, consumption of multivitamins, which included vitamins A, B_1_, B_2_, B_12_ and D, reduced the risk of obesity in children and adolescents^(15)^. High prevalence of vitamin A, B_1_, vitamin C and vitamin D deficiency, with deficiency in vitamin D the highest among all vitamins, was found in obese adults^(16)^. Additionally, overweight and obese adults have baseline serum vitamin D that correlated negatively with their BMI^(17)^, and they generally consume energy dense, nutrient poor diet^(18)^. Based upon the above reports, the following sections, therefore, focus on vitamins that were reported to be decreased or deficient in obese children and adult, namely vitamins A, B_1_, C and D, highlighting evidences of the correlation between these vitamins and abdominal adiposity, lipid profile and inflammation, and providing evidences of the effects of vitamin supplementation on obesity and obesity-related metabolic status.

### Vitamin C

All overweight and obese patients have vitamin C status that correlated inversely with BMI, and serum vitamin C levels were significantly lower in obese than overweight patients. Moreover, serum vitamin C levels were lower in individuals who have hypertriglyceridaemia and low levels of high-density lipoprotein-cholesterol (HDL-C) as compared with those having normal triglyceride and HDL-C levels. Low vitamin C status was the independent risk factor for obesity and lipid metabolic dysfunction^(19)^. However, the effects of vitamin C on lipid metabolism, and vitamin C supplementation on lipid profile, are unclear. In rodent, vitamin C was found to reduce visceral obesity through activating peroxisome proliferator-activated receptor-α (PPARα)^(20)^. But human studies on the effects of vitamin C on adipocytes, including differentiation, triglyceride accumulation and lipolysis inhibition are conflicting^(21)^, as detailed below.

A meta-analysis of 13 RCTs indicated that a minimum 4 weeks of at least 500 mg daily vitamin C supplementation can significantly decrease serum low-density lipoprotein-cholesterol (LDL-C) and triglyceride concentrations^(22)^. However, participants in those RCTs were not obese but were either healthy, elderly, diabetic or have hyperlipidaemia. Another meta-analysis of RCTs conducted subsequently to ascertain the effects of vitamin C on blood total cholesterol, LDL-C, HDL-C and triglyceride concluded that vitamin C supplementation has no significant effect on lipid profile^(23)^. Although vitamin C supplementation did not change blood lipids concentrations significantly, it has benefited specific groups such as lowering the total cholesterol of younger participants, the LDL-C of healthy participants and the triglyceride of diabetics while increasing the HDL-C in diabetics. The authors, therefore, concluded that vitamin C supplement provided more benefit to individuals with higher baseline levels of total cholesterol and triglyceride^(23)^. Nevertheless, similar to the earlier meta-analysis^(22)^, the adult participants in the study were non-obese.

It appears that patients who have altered metabolic parameters require high dose and chronic consumption of vitamin C if they were to gain any potential benefits of vitamin C. This is due partly to the fact that vitamin C does not accumulate in the body and the excess is eliminated immediately through urine^(24)^. Diabetic patients administered 1000 mg daily oral vitamin C for 4 months showed improvement in whole body glucose disposal and non-oxidative glucose metabolism^(25)^, but 800 mg daily intake of vitamin C for 4 weeks did not improve glucose metabolism or insulin resistance^(26)^. This means that if the dose of vitamin C is insufficient to fully replenish the low baseline level of vitamin C in these patients^(27)^, it is ineffective in improving endothelial dysfunction and insulin resistance. The same condition may apply to obesity.

In obesity, white AT overgrowth leads to increased production of pro-inflammatory cytokines including TNF-α, interleukin-6 (IL-6), MCP-1 and inducible nitric oxide synthase (iNOS), activation of inflammatory pathways of IкB kinase (IKK-β) and nuclear factor-кB (NF-кB), mitochondrial dysfunction, reactive oxygen species (ROS) overproduction and depletion of the antioxidant defense^(28)^. Oxidative stress has been associated with low grade chronic inflammation and insulin resistance^(28)^. While it might not be easy to demonstrate the causal effect of the relationship, it is believed that inflammation occurs during the early stage of obesity development, followed by an enhanced oxidative stress status^(13,29)^. Therefore, dietary supplements which have anti-inflammatory and/or antioxidant properties maybe beneficial in the management of metabolic derangement in obesity.

Vitamin C is an antioxidant due to its ability to donate electron. In isolated rat adipocytes, vitamin C reduced intracellular and extracellular ROS production^(28)^. *In vitro*, vitamin C inhibited the activation of NF-кB signalling^(30)^ and IKK-β enzyme^(31)^. *In vitro* and *in vivo* animal studies showed that vitamin C increased the production of lipoxin A4, an anti-inflammatory and antioxidant^(32)^. Broiler chicks supplemented with a vitamin C-rich diet have decreased hepatic mRNA expressions of IL-1β, IL-6, interferon-γ, Toll-like receptor-4 and heat shock protein 70 compared with those fed with a control diet. Lipid peroxidation in the serum and liver of these animals was also significantly decreased^(33)^. Data of the abovementioned effects of vitamin C supplementation on human, however, are not available, and Abdali *et al.*^(34)^ thought that vitamin C is of marginal benefits to obese and diabetic patients.

### Vitamin B_1_ (thiamine)

Thiamine is a water-soluble vitamin that is not stored substantially in the body. Many morbidly obese patients are thiamine-deficient, and the prevalence of thiamine deficiency was 7–8 folds higher in African Americans and Hispanics than in Caucasian^(35)^. The reason for racial differences is unknown. Thiamine catalyses several key biochemical reactions involved in glucose metabolism. This means that metabolism of a sugar-high diet requires high amount of thiamine. Obese patients who consume energy dense and high sugar content diet may, therefore, have much higher thiamine needs^(36)^, and accelerated thiamine depletion during glucose metabolism. Thiamine deficiency leads to impairment in insulin synthesis and secretion^(37)^. Severe deficiency is associated with increased endothelial nitric oxide synthase (eNOS) production, intercellular adhesion molecule-1 (ICAM-1) levels and ROS production^(38)^.

Thiamine has antioxidant properties. *In vitro* studies showed that thiamine supplementation inhibited lipid peroxidation^(39)^, and limited hyperglycaemia-induced von Willebrand factor secretion from bovine aortic endothelial cells thereby endothelial cell dysfunction^(40)^. Given the high rates as well as the consequences of thiamine deficiency in obese and diabetic patients, Via^(16)^ opined that thiamine supplementation may be considered in this group of people. However, to date, there is no published data on thiamine requirements in overweight and obese patients, i.e. how much thiamine should patients consume in order to tackle the consequences resulting from thiamine deficiency is unclear. Several reasons might explain this: (1) the ability of a thiamine-rich diet in causing weight loss in obese and overweight individuals has not been demonstrated^(41)^; (2) the lack of studies and evidence of the effectiveness of long-term thiamine supplementation on combating the metabolic changes in obesity; (3) the amount of thiamine needed by obese people may simply be too high since they have high sugar level contributed by insulin resistance and/or energy dense diet, which accelerate thiamine usage, but they already have a low thiamine level in the body, therefore making the approach to increase thiamine level through diet and supplement non-feasible; (4) the beneficial effects of thiamine may be confounded by the body sugar levels; and (5) there is other dietary vitamin that is superior to thiamine in managing body weight, and glucose and lipid metabolism.

### Vitamin D

The main function of vitamin D is for the maintenance of bone tissue, and homeostasis of calcium and phosphorus. Extra-skeletal functions of vitamin D include those on the muscular, insulin sensitivity and immune system, to name a few^(42)^. Moderate vitamin D deficiency leads to increased bone turnover and a greater risk of bone fractures, while severe deficiency causes osteomalacia in adults and rickets in children. Vitamin D deficiency is also associated with visceral adiposity-related metabolic syndrome such as obesity, dyslipidemia, insulin resistance, diabetes, cardiovascular diseases and hypertension^(43)^. Serum vitamin D status correlates negatively with the BMI of children, adolescents and adults^(15,17)^.

The possible causes for the decreased vitamin D serum level in obesity have been reported in numerous review articles^(43–46)^, although as cited in these reviews, the studies of some of those causes were conflicting. The more probable causes of decreased vitamin D in obesity are: (1) vitamin D sequestration in AT – as the amount of AT increases, vitamin D is accumulated and retained in AT, resulting in a lower plasma concentration^(47)^; (2) low sunlight exposure^(46)^ and (3) altered volumetric dilution of vitamin D – vitamin D is distributed into serum, muscle, fat and liver, in which all these compartments are increased in obesity^(48)^. These mechanisms suggest that weight loss or reducing visceral adiposity by increasing physical activity for example, and exposure to sunlight will increase circulatory levels of vitamin D. If so, do obese people still need vitamin D supplementation?

A 26 weeks treatment with 7000 IU (175 μg) vitamin D daily did not change body fat accumulation of obese patients as compared to placebo^(49)^. In a 12 months RCT, obese patients receiving 2000 IU vitamin D daily and lifestyle-based weight loss programme did not have significant reduction in body weight, body composition, BMI, insulin and C-reactive protein (CRP) levels when compared with the placebo group who received lifestyle-based weight loss programme only^(50)^. In the present study^(50)^ however, the placebo group also did not have significant changes in body weight and body composition between pre-treatment and at 12 months, and possibly because of this, according to the above proposed mechanisms (1) and (3), the group has no significant change in serum vitamin D at 12 months (20 ng/ml). The study group that received vitamin D supplementation has significant elevation in serum vitamin D concentration (35 ng/ml), suggesting that intake of 2000 IU once daily for 12 months is sufficient to increase serum vitamin D in obese patients. Together with the study by Wamberg *et al.*^(49)^, present evidences do not support vitamin D supplementation in reducing body weight and body composition.

The effects of vitamin D on adipogenesis and lipid accumulation contradict between *in vitro* studies *per se* and between *in vitro* and *in vivo* studies^(44)^. *In vitro* studies using mouse cell lines showed that vitamin D inhibited adipogenesis by down-regulating CCAAT enhancer binding proteins (C/EBP) and PPARγ, and inhibited lipid accumulation by suppressing the expression of sterol regulatory element binding protein 1c (SREBP1c) and LPL. SREBP1c is a protein that promotes the expression of genes involved in glucose metabolism, lipogenesis and fatty acid production. In mouse primary cell culture, however, vitamin D was pro-adipogenic. Pro-adipogenic effects of vitamin D were also observed in *in vivo* animal models. To date, there is no evidence to suggest that vitamin D supplementation suppresses adipogenesis signalling pathways in human. Studies of the effects of vitamin D on lipid profiles of obese patients are available but have reported conflicting results – a placebo-controlled RCT showed that vitamin D supplement has no effect on patients’ plasma triglyceride, HDL-C and total cholesterol levels^(49)^. But in a meta-analysis, vitamin D treatment increased HDL-C and oral glucose insulin sensitivity and decreased triglyceride but it also increased LDL-C^(51)^.

In terms of the anti-inflammatory effects of vitamin D, Wamberg *et al.*^(49)^ reported that the consumption of 7000 IU vitamin D daily for 26 weeks did not significantly decrease circulatory inflammatory markers such as CRP, IL-6, MCP-1, leptin and adiponectin. In other studies, intake of 40 000 IU vitamin D weekly for one year decreased serum IL-6 but increased CRP, and demonstrated no effects on insulin resistance, TNF-α^(52)^, ICAM-1, interferon-γ, MCP-1 and CRP^(53)^ in overweight and obese patients. Despite these contradictory findings, since patients with BMI above 38⋅4 kg/m^2^ have baseline vitamin D that correlated negatively with serum levels of leptin and resistin, and positively with serum adiponectin levels, some researchers suggest consuming vitamin D to improve AT function and prevent obesity-related diseases^(54)^, and this recommendation may be relevant to morbidly obese patients. However, if the mechanisms that cause serum vitamin D reduction in obesity are as reported in the literature, any means of reducing body weight would increase serum vitamin D. Moreover, there are very few vitamin D-rich sources, and the amount of vitamin D from the same source but of different origin could vary and could be relatively low^(55)^.

### Vitamin A

Dietary vitamin A was found to be associated significantly with plasma retinol levels^(56)^, while inadequate intake is the major factor contributing to vitamin A deficiency^(57)^. Overweight and obese individuals who were deficient in serum levels of vitamin A and vitamin D, were said to have high calorie malnutrition^(17)^. The BMI of obese adults and children correlated negatively with serum vitamin A levels^(56–58)^. A significantly lower serum vitamin A concentration in obese children than that of normal weight children was also associated with increased waist circumference, fasting plasma glucose and triglyceride, and decreased HDL-C^(56)^. Research to understand the effects of vitamin A on obesity has been done extensively on its precursor, carotenoids. This may be explained by pre-clinical and clinical studies, which indicated that carotenoids and carotenoids derivatives prevented abdominal adiposity, and have anti-inflammatory and antioxidant effects. Moreover, intact carotenoid molecules and carotenoid cleavage products may have additional biological activities whose relevance for human health are still unknown^(59)^.

There are over 600 carotenoids present in nature, of which some 50 are found in human diet, mainly fruits and vegetables. But only about half of those found in the diet are detected in human blood and tissue^(60,61)^. The most abundant carotenoids in human serum are α-carotene, β-carotene, lycopene, lutein, zeaxanthin and β-cryptoxanthin^(61)^. Retinol may be generated *de novo* from α-carotene, β-carotene and β-cryptoxanthin^(59)^. Carotenoids are accumulated mainly in the liver and adipose tissues^(62)^. The tissue distribution of carotenoids implies their potential effects on the metabolic processes within these tissues^(63)^.

Carotenoids are lipid-soluble and widely found in plant-based sources. Besides fruits and vegetables, carotenoids are available in commonly consumed food such as bread, eggs, milk, beverages, fats and oils^(64)^. Carotenoids insufficiency was reported in adults and children with obesity^(65)^. A study by Harari *et al.*^(66)^ showed that all the five serum carotenoids measured (α-carotene, β-carotene, lutein, lycopene and ζ-carotene) were significantly lower in adult obese patients compared with non-obese individuals. These obese patients have higher total body fat and central fat, but lower HDL-C, and reduced insulin sensitivity. Similar to adults, serum carotenoids (α-carotene and β-carotene) of obese children correlated negatively with BMI, waist circumference, fat mass and triglyceride, and positively with HDL-C^(67)^. In healthy adults, serum total and specific carotenoid concentrations were associated inversely with CRP, MCP-1 and TNF-α^(68)^. In school-aged children, plasma β-carotene was associated negatively with plasma IL-6 levels^(69)^. Obese individuals who have significantly lower circulating carotenoids than healthy subjects may, therefore, be exposed to a higher risk of inflammation.

A recent meta-analysis^(65)^ reported that carotenoids supplementation in overweight and obese individuals might contribute to the reduction in body weight, BMI, waist circumference and total cholesterol while increasing HDL-C. However, carotenoids have no effect on fat ratio, LDL-C and triglyceride concentrations. Among the six carotenoids present abundantly in serum, only α-carotene, β-carotene and β-cryptoxanthin have pro-vitamin A activity. The importance of the other carotenoids in human nutrition is rather limited^(70)^. Because β-carotene oxygenase 1 is the sole enzyme responsible for the conversion of carotenoids to vitamin A, significant interest, hence research has been conducted on β-carotene to delineate its mechanism of action on adipogenesis and inflammation.

**Fig. 1.**
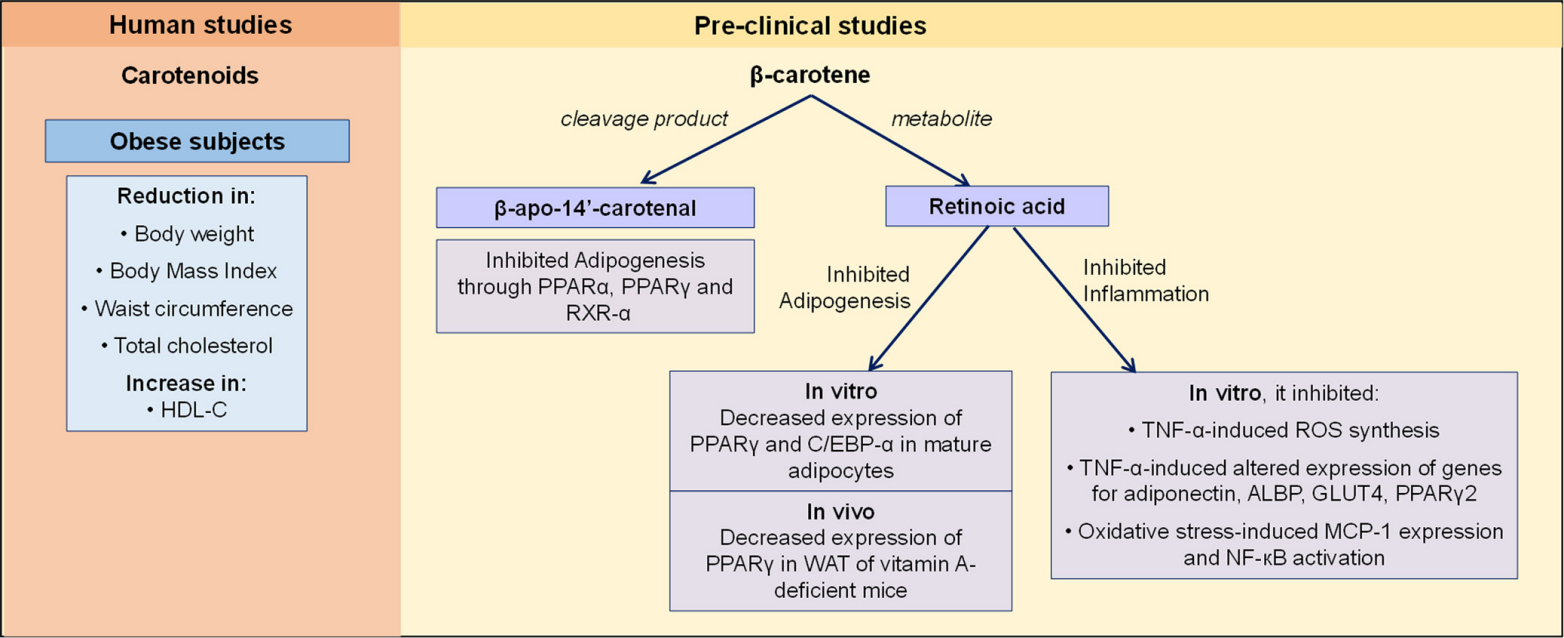
The effects of carotenoids and β-carotene on obesity. In human studies, carotenoids were reported to improve weight, body composition and HDL-C in obese subjects. In pre-clinical studies, β-carotene's cleavage product, β-apo-14′-carotenal inhibited adipogenesis through suppressing PPARα, PPARγ and RXR-α activation. The metabolite of β-carotene, retinoic acid was reported to inhibit adipogenesis and inflammation. These effects were seen in *in vitro* and *in vivo* studies. ALBP, adipocyte lipid-binding protein; C/EBP-α, CCAAT enhancer binding proteins; GLUT4, glucose transporter-4; HDL-C, high-density lipoprotein-cholesterol; MCP-1, macrophage chemoattractant protein-1; NF- кB, nuclear factor-кB; PPARα, peroxisome proliferator-activated receptor-α; PPARγ, peroxisome proliferator-activated receptor-γ; ROS, reactive oxygen species; RXR-α, retinoid X receptor-α; TNF-α, tumor necrosis factor-α; WAT, white adipose tissue.

*In vitro* and *in vivo* animal studies showed that β-carotene through its cleavage product, β-apo-14′-carotenal, inhibited adipogenesis through repression of PPARα, PPARγ and retinoid X receptor-α activation^(71)^. β-carotene through its metabolite retinoic acid, decreased the expression of PPARγ and C/EBP-α, and the lipid content of mature adipocytes. β-carotene administration, through increasing retinoic acid signalling, down-regulated PPARγ expression in white AT of vitamin A-deficient mice^(72)^. β-carotene exhibited anti-inflammatory effects in adipocytes by limiting TNF-α-induced ROS production as well as alterations in the expression of genes related to insulin sensitivity, including adiponectin, adipocyte lipid-binding protein, glucose transporter-4, PPARγ2 and adiponectin protein^(73)^. Also in adipocytes, β-carotene inhibited oxidative stress-induced adiponectin dysregulation, increased MCP-1 expression and NF-кB activation^(74)^ (Fig. 1). As to whether the effects of anti-adipogenesis and anti-inflammation of β-carotene are seen in human have not been established yet.

## Conclusions

A nutritional-balanced diet is especially important for obese individuals, to maintain health and support pharmacotherapies and other lifestyle modification strategies. Vitamin supplementation that takes into consideration all of the following criteria may lead to a better treatment outcome. The compound is distributed widely in food sources, cheap and easily accessible; is lipid-soluble, which will give better tissues bioavailability than water-soluble compounds; has been shown to correlate negatively with body weight and composition and has demonstrated anti-adipogenic, anti-inflammatory and antioxidant properties from RCTs. The treatment target may be on normalising the decreased serum levels of vitamins in obese patients.

**Fig. 2.**
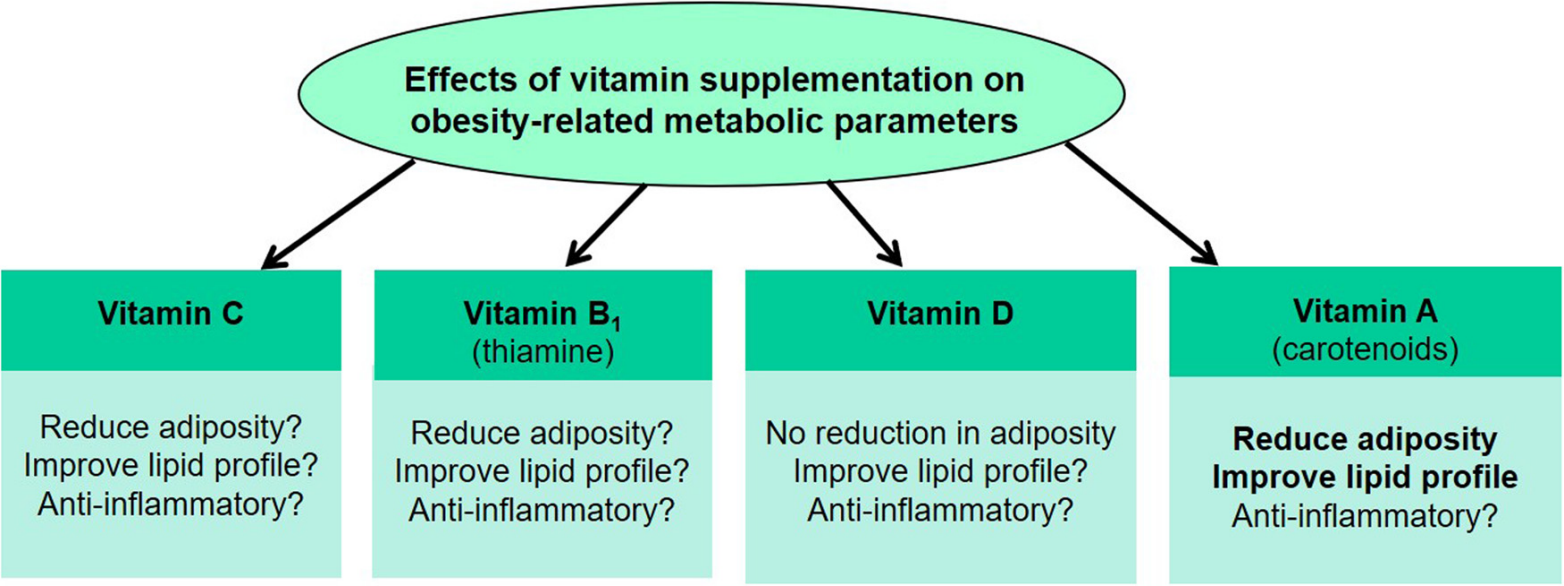
Summary of the effects of vitamins A–D supplementation on metabolic changes in obesity. Dietary vitamins including vitamins C, B_1_ and D have either conflicting evidences or no evidence of reducing adiposity, improving lipid profile and the inflammatory status of obese subjects. The precursor of vitamin A, carotenoids, reduce adiposity and improve lipid profile. β-carotene was reported to have anti-inflammatory effects in pre-clinical studies but such evidence is lacking in human studies.

There is a lack of evidence that supports any beneficial effects of vitamin C supplementation on glucose and lipid metabolism, and suppression of inflammation in obese patients. However, as an antioxidant, long-term and high dose vitamin C consumption may provide some benefits to the overall health. Thiamine, despite being an antioxidant, requires more human intervention studies to determine its benefits as well as feasibility in supporting obesity treatment. Although vitamin D may have some anti-inflammatory effects that improve AT functions, such evidence, together with evidences of its effects on body weight reduction and adipogenesis, are neither clear nor convincing. Therefore, vitamin D may not be the first line vitamin supplementation for obesity treatment. As compared with vitamins C, B_1_ and D, vitamin A and its precursor carotenoids, specifically β-carotene, appeared to be the more promising vitamin that can be used for the regulation of body weight, lipid metabolism and inflammatory status in obesity (Fig. 2). However, human study is warranted to confirm the anti-adipogenic, anti-inflammatory and antioxidative effects seen for β-carotene in *in vitro* and *in vivo* animal studies.

Future human intervention studies of carotenoids and β-carotene should investigate not only their effects on visceral adiposity and AT functions, but the lowest effective doses that produce health benefits. This is because dietary intervention may be implemented long-term, and as such any potential dose-dependent side effects of carotenoids should be minimised. Effective management of obesity through dietary vitamin that goes along with drug, physical and behavioural therapies, may also reduce the risk of T2DM development in obesity.
